# Genome Sequences of Burkholderia thailandensis Strains E421, E426, and DW503

**DOI:** 10.1128/MRA.00312-20

**Published:** 2020-05-21

**Authors:** Catherine M. Mageeney, Anupama Sinha, Kelly P. Williams, Steven S. Branda

**Affiliations:** a Sandia National Laboratories, Livermore, California, USA; University of Delaware

## Abstract

We present the draft genome sequences of three Burkholderia thailandensis strains, E421, E426, and DW503. E421 consists of 90 contigs of 6,639,935 bp and 67.73% GC content. E426 consists of 106 contigs of 6,587,853 bp and 67.73% GC content. DW503 consists of 102 contigs of 6,458,767 bp and 67.64% GC content.

## ANNOUNCEMENT

Burkholderia thailandensis, occurring naturally in soil and water, serves as a surrogate for the study of its more pathogenic congeners, select agent bacteria Burkholderia pseudomallei and Burkholderia mallei, with which it shares many genome features and pathogenesis mechanisms, and it provokes similar immune responses in mammalian systems (1). We report the genome sequences of three B. thailandensis strains obtained from BEI Resources (Manassas, VA, USA). B. thailandensis E421 and E426 were isolated independently from a rice field in Northeast Thailand. B. thailandensis DW503 is an allelic exchange mutant of B. thailandensis E264 in which the gene cluster comprising *amrR*, *amrA*, *amrB*, and *oprA* is deleted (2).

E421, E426, and DW503 were grown in Luria broth in Erlenmeyer flasks at 37°C for ∼16 h. From each culture, 2 ml was centrifuged at 4,000 × *g* for 4 min, and DNA was extracted from the pelleted bacteria using the DNeasy blood and tissue kit (Qiagen, Hilden, Germany). DNA sequencing libraries were generated using the Nextera DNA library prep and DNA sample preparation index kits (Illumina, San Diego, CA, USA). Library concentrations were measured using the Qubit fluorometer with its high-sensitivity DNA assay kit (Thermo Fisher Scientific, Waltham, MA, USA). Equal amounts of the three libraries were mixed to generate a multiplexed library; its concentration was measured using the Qubit device, and fragment sizes were measured using the Bioanalyzer electrophoresis system with its high-sensitivity DNA analysis kit (Agilent, Santa Clara, CA, USA). The multiplexed library was sequenced in paired-end mode using a NextSeq 500 instrument with its high-output (300 cycles) kit (Illumina).

Reads were quality filtered using BBDuk (27 June 2016 release with the following parameters: ktrim = r, k = 21, mink = 11, hdist = 1) (http://jgi.doe.gov/data-and-tools/bb-tools/). Assembly of the sequencing reads was completed using the default settings for SPAdes v. 3.9.0 (3) (Table 1). FastANI alignment (4), run using default parameters, revealed that E421 and E426 share 99.8% average nucleotide identity (ANI) with E264. DW503 shares 99.9978% ANI with E264; its deletion at nucleotide position 129447 (GenBank accession number JAAAQG010000016) spans 148 kbp (CP000086.1, coordinates 2668007 to 2816380) and 99 genes (chromosomal loci BTH_I2364 to BTH_I2461), including the gene cluster comprising *amrR*, *amrA*, *amrB*, and *oprA*, as confirmed by analysis of the raw reads with readStepper (5). Sequence annotation was performed using our tater.pl pipeline (8), which calls Prokka v. 1.11 (7), tFind, and rFind software (8); counts for coding regions, miscellaneous RNA (miscRNA), rRNA sequences, and tRNA sequences (6) are listed in Table 1. All the strains had hits to the following Rfam (9) miscRNA profiles: 6S, Bacteria_large_SRP, Bacteria_small_SRP, Betaproteobacteria_toxic_sRNA, *cspA*, FMN, Intron_*gpI*, mini-*ykkC*, P9, *pfl*, RNaseP_bact_a, SAH_riboswitch, SECIS_3, *sucA*, and TPP. In addition, E421 and E426 had hits to C4, cobalamin, and *isrK*. Additionally, sequences were analyzed to identify CRISPR-Cas arrays (CRISPRCasFinder [10]), prophages, and other genomic islands (6, 8). The TIGER package (6), including tater.pl, genomic island prediction software, and prophage calling software, is available at github.com/sandialabs/TIGER.

**TABLE 1:** Assembly statistics and genome sequence features

Feature^*a*^	Data for strain:		
	E421	E426	DW503
No. of reads	133,200,415	136,919,906	61,127,893
Coverage (fold)	2,824	2,997	1,440
No. of contigs	90	106	102
Total size (bp)	6,639,935	6,587,853	6,458,767
*N*_50_ (bp)	159,164	121,401	156,901
GC content (%)	67.73	67.73	67.64
No. of ambiguous bases	10	924	0
No. of CDS	5,608	5,524	5,427
No. of rRNA operons	3	2	2
No. of tRNA/tmRNA genes	52	47	50
No. of miscRNA genes	27	25	21
No. of CRISPR-Cas arrays	1	3	4
No. of GIs	7	5	8
Prophage coordinates^*b*^	**45oatA|selB** 02:159872–204745	**41Hyp** 04:49998–75818	**16mutS** 12:52204–68122
	**17mutS** 10:52214–68931		**44pcaK** 33:9582–54013
	**40F** 16:5517–45277		**11R** 49:24269–32704, 84:10537–2253
Other GI coordinates^*b*^	**26dapA|sbp** 04:49955–75760	**8G** 01:381563–389876	**49CbiA|HBT** 08:149573–198632
	**33T** 19:84650–117520	**3Phage_GPD** 14:8710–49502	**19Hyp** 20:23740–42637
	**22S** 21:23808–45519	**19A** 16:4992–8027	**22A** 55:4198–4233, 68:1–10395, 82:2548–1, 89:1–1497, 55:1–4200
	**24P** 32:39753–64195	**26dapA|sbp** 23:49376–68235	**48R** 50:2569–32098, 89:1–1497, 67:1–10868, 83:2372–1, 50:2517–2568
			**3Acyl_transf_3** 42:53140–55361, 05:2308–2523, 17:137203–136908
GenBank accession no.	JAAAQH000000000	JAAAWT000000000	JAAAQG000000000
NCBI SRA project no.	PRJNA600278	PRJNA600279	PRJNA600280
NCBI run no.	SRR11144399	SRR11142218	SRR11073226

a CDS, coding DNA sequences; tmRNA, transfer-messenger RNA.

b Names of prophages and other genomic islands (GIs) are in bold, followed by their corresponding contig numbers and nucleotide positions (note that DW503 GIs 11R, 22A, 48R, and 3Acyl_transf_3 are split between two or more contigs).

Names of prophages and other genomic islands (GIs), contig numbers, and coordinates are listed in Table 1. DW503 is a deletion mutant of E264 (GenBank assembly number GCA_000012365) (2); we previously reported the coordinates for E264 prophages and GIs (6). Some of the GIs in E264 were found to be split between two or more DW503 contigs (separated by commas in Table 1).

### Data availability.

The assemblies have been deposited at GenBank under the following accession numbers: JAAAQH000000000 (E421), JAAAWT000000000 (E426), and JAAAQG000000000 (DW503). The raw data have been deposited at the NCBI Sequence Read Archive (SRA) under the BioProject and run accession numbers PRJNA600278 and SRR11144399 (E421), PRJNA600279 and SRR11142218 (E426), and PRJNA600280 and SRR11073226 (DW503), respectively.
